# Common Polymorphisms in the 5-Lipoxygenase Pathway and Risk of Incident Myocardial Infarction: A Danish Case-Cohort Study

**DOI:** 10.1371/journal.pone.0167217

**Published:** 2016-11-28

**Authors:** Anders Gammelmark, Michael S. Nielsen, Søren Lundbye-Christensen, Anne Tjønneland, Erik B. Schmidt, Kim Overvad

**Affiliations:** 1 Department of Cardiology, Aalborg University Hospital, Aalborg, Denmark; 2 Department of Clinical Medicine, Aalborg University, Aalborg, Denmark; 3 Unit for Clinical Biostatistics and Bioinformatics, Aalborg University Hospital, Aalborg, Denmark; 4 Danish Cancer Society Research Center, Copenhagen, Denmark; 5 Section for Epidemiology, Department of Public Health, Aarhus University, Aarhus, Denmark; Johns Hopkins University, UNITED STATES

## Abstract

**Background:**

The 5-lipoxygenase pathway (5-LOX) has been implicated in the development of cardiovascular disease and studies have suggested that genetic polymorphisms related to key enzymes in this pathway may confer risk of myocardial infarction (MI). This study investigated the association of pre-selected genetic polymorphisms in four candidate genes of 5-LOX (arachidonate 5-lipoxygenase and its activating protein (*ALOX-5* and *FLAP*), leukotriene A4 hydroxylase (*LTA4-H*) and leukotriene C4 synthase (*LTC4-S*)) with incident MI.

**Methods:**

In a Danish cohort including 57,053 participants, aged 50–64 at enrolment and recruited from 1993–97, we conducted a case-cohort study including cases with incident MI and a randomly selected sub cohort of 3,000 participants. Cases were identified from national registries through July 2013. A total of 22 SNPs were selected and genotyped using the commercially available KASP^™^ assay. A tandem-repeat polymorphism, located in the *ALOX-5* gene, was genotyped by multi-titre plate sequencing. Haplotypes were inferred using PHASE 2.1.

**Results:**

During a median follow-up of 17.0 years we identified 3,089 cases of incident MI. In *FLAP*, two SNPs were negatively associated with incident MI (rs9551963 & rs17222842) while one SNP (rs2247570) located in *LTA4-H*, was associated with higher risk of MI when comparing subjects with two copies of the variant allele to homozygotes for the wild type. However, only rs17222842 remained significantly associated with MI after correcting for multiple testing. Furthermore, the promoter polymorphism rs59439148 was associated with risk of MI in men. For male carriers of two variant alleles we found a hazard ratio of 1.63 (95% CI: 1.06;2.52) compared to homozygotes for the wild type. Previously described haplotypes (Hap-A -B, -E and -K) were not associated with MI in our population.

**Conclusion:**

In conclusion, some common polymorphisms in the 5-lipoxygenase pathway were modestly associated with incident MI, suggesting a potential role for this pathway in the development of cardiovascular disease.

## Introduction

**Fig 1:**
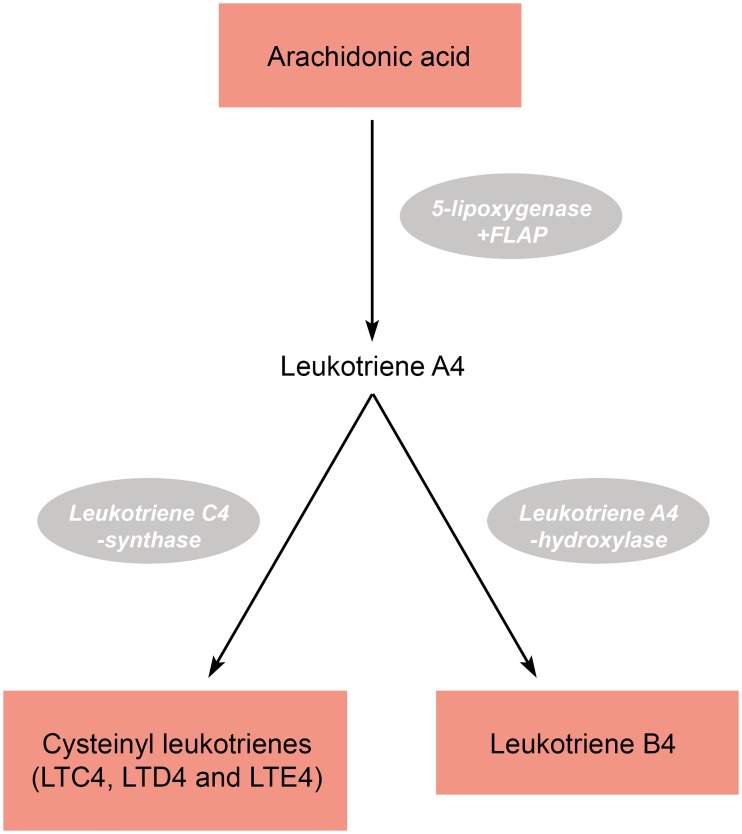
Schematic outline of the 5-LOX pathway leading to the formation of leukotrienes. The figure gives a schematic overview of the formation of 4-series leukotrines from arachidonic acid. The first step is catalysed by 5-lipoxygenase and 5-lipoxygenase activating protein (FLAP), which is also the rate-limiting step in the pathway. Next, leukotriene A4 is rapidly metabolised to either leukotriene B4 or the cysteinyl leukotrienes by leukotriene A4-hydroxylase or leukotriene C4-synthase, respectively.

Atherosclerosis is a multifactorial disease involving both environmental and genetic factors. In recent years, focus has turned on the complex cascade of inflammatory processes that takes place in the vessel wall and within atherosclerotic plaques [1,2]. In this context the 5-lipoxygenase (5-LOX) pathway has received attention. Thus, the 5-LOX pathway metabolizes arachidonic acid (AA), leading to the formation of highly pro-inflammatory lipid mediators called leukotrienes (LTs) [3]. The 5-LOX pathway consists of four key enzymes, where arachidonate 5-lipoxygenase (ALOX-5) and the ALOX-5 activating protein (FLAP) constitutes the first enzymatic step followed by conversion to either leukotriene C4 synthase (LTC4-S) or leukotriene B4 hydroxylase (LTB4-H) resulting in the formation of either cysteinyl leukotrienes or B-series leukotrienes, respectively (Fig 1).

Evidence at multiple levels, including animal, biochemical and human studies, has linked this pathway and the bioactive LTs to the development of atherosclerosis and athero-thrombotic disease [3–7]. Thus, in a mouse model, it was demonstrated that the knock out of the *ALOX-5* gene led to a high resistance against the development of atherosclerosis [8]. Other aspects of the 5-LOX pathway have been implicated in atherosclerosis traits in animal studies, including the leukotriene B4 –receptor [9,10] and the *FLAP* gene [11]. Furthermore, studies on human atherosclerotic plaques have identified the presence of 5-LOX enzymes and levels of ALOX-5 were higher in the more advanced plaques [12]. In addition, high levels of ALOX-5 and LTA4-H in human plaques have been associated with symptoms of plaque instability [13,14], suggesting a key role of the 5-LOX pathway in late stages of atherosclerosis and atherothrombotic events.

A number of epidemiological studies have examined four candidate genes, encoding the enzymes involved in the 5-LOX pathway. Most attention has been focused on *ALOX-5* and *FLAP*, where Dwyer et al. examined a tandem repeat polymorphism in the promoter region of *ALOX-5* that was associated with higher intima-media thickness of the carotid arteries, a marker of atherosclerosis, when comparing carriers of the variant allele with homozygotes of the wild type allele [15]. This polymorphism has been investigated in a number of studies with different endpoints, including ischemic stroke and MI [16–20], but the results have been conflicting.

In a genome-wide association study, the deCODE investigators identified *FLAP* as an important gene involved in atherothrombotic disease and reported two haplotypes (Hap-A and Hap-B) that were associated with higher risk of MI and stroke [21,22]. Some studies have supported these findings [23–27] while others did not find these haplotypes to be associated with risk of MI or stroke [19,28–30].

The deCODE investigators also defined a risk haplotype (Hap-K) for MI [31], covering the *LTA4-H* gene which was confirmed by other investigators [19,32]. Following these studies, Zhao et al. defined a new haplotype (Hap-E) that was associated with a lower risk of MI among carriers compared to non-carriers [30].

Finally, two promoter polymorphisms have been identified in *LTC4-S* and investigated in three studies, with conflicting results [33–35].

Thus, from previous studies on four candidate genes, encoding key enzymes in the 5-LOX pathway, it has been suggested that genetic variants may be associated with atherosclerotic disease. Following a review of the literature, we selected 22 SNPs from these four candidate genes to investigate the association with incident MI in a large Danish cohort study.

## Materials and Methods

### Study design and population

The Danish Diet, Cancer and Health study is a prospective cohort study, which has been described in detail previously [36]. Briefly, 160,725 persons aged 50–64 years were invited between December 1993 and May 1997. Eligible participants were born in Denmark, living in the urban areas of Copenhagen and Aarhus and not registered with a cancer diagnosis in the Danish Cancer Registry at the time of invitation. If a cancer diagnosis was found that was not already recorded in the Cancer Registry at time of invitation, participants were excluded in line with the intention-to-include criteria. Further, participants registered with a previous MI or cardiac arrest were excluded as well. At baseline, each participant filled in a detailed questionnaire on diet, lifestyle, socio-economic status and medical history. Blood and adipose tissue samples were collected.

For the present study we used a nested case-cohort design including all cases with incident MI and a randomly selected sub cohort (*n* = 3,000) to represent the cohort. The study was conducted in accordance with the Helsinki Declaration and all participants provided written informed consent. The study, including the consent procedure, was approved by The Regional Ethics Committee, North Denmark Region (approval number, N-20140071).

### Selection and genotyping of SNPs

From a review of the current literature, we selected four candidate genes to examine the 5-LOX pathway (*ALOX-5*, *FLAP*, *LTA4-H* and *LTC4-S*). Next, 22 SNP markers were selected based on previous reported associations with cardiovascular disease, with preference for coronary artery disease, and a confirmed minor allele frequency (MAF) > 0.05 in Caucasians.

From whole blood, DNA was extracted using Kleargene^™^ XL DNA extraction kit (LGC Genomics, Queens Road, Teddington, Middlesex, UK). Next, contaminants were removed by washing and DNA was subsequently eluted into a low salt buffer. Extracted DNA were stored at -20°C.

SNP genotyping was performed by LGC Genomics using the commercially available KASP^™^ genotyping assay. KASP is based on a competitive, allele specific PCR genotyping technique with a homogenous fluorescent based reporting system [37]. The reaction mix was aliquoted to standard 96-well plates containing DNA-samples from the study cohort, including at least one "no template control" per plate. PCR was performed and the fluorescent signal was analysed using a BMG PHERAstar plate reader (BMG Labtech Ltd., Aylesbury, UK). The analysis was performed according to the protocol provided by LGC Genomics [38]. SNP alleles correspond to the positive/forward DNA-strand according to dbSNP, human assembly GRCh38.p2 [39].

### Genotyping of *ALOX-5* tandem repeat polymorphism

The tandem repeat polymorphism was analysed by microtitre plate (MTP)-sequencing technique, using standard 96-well plates. PCR-products were prepared from genomic DNA, using MyTaq^™^ DNA polymerase (Bioline US Inc.) along with the following primers: 5’-TCAGGAGAGAACGAGTGAAC-3’ (forward) and 5’-GTCCAGGTGTCCGCATC-3’ (reverse). 40 reaction cycles were performed at 55°C. From the PCR-products, sequencing was done using an ABI 3730XL DNA analyser (Thermo Fischer Scientific Inc.) and Chromatograms were interpreted by a trained laboratory technician, identifying the number of tandem-repeats for each allele.

### Outcome assessment

We identified all participants in the cohort who were registered with an incident diagnosis of MI in the Danish National Patient Registry and/or the Danish Causes of Death Registry, according to the International Classification of Disease (ICD) 8 (410.00–410.99) or ICD-10 (I21.0-I21.9) coding, during the study period. Furthermore, all cases of cardiac arrest (ICD-8: 427.27 or ICD-10: I46.0-I46.9) were included if the arrest was considered to be of cardiac origin after validation in each individual case. An earlier study from our Department validated the MI diagnosis from baseline through 2003 by complete review of all medical records and found a positive predictive value above 92% when the diagnoses were obtained from a hospital ward [40]. All validated cases of MI from this validation study were included as cases for the present study. From January 2004 through July 2013 all participants with an incident MI diagnosed from a ward were readily accepted as cases without further validation. All other diagnoses of incident MI and cardiac arrest were validated by reviewing a complete list of diagnoses and interventional procedures recorded in the Danish National Patient Registry for each potential case.

### Statistics

Allele frequencies were tested for Hardy—Weinberg equilibrium (HWE) in the sub cohort using a chi-square test (*X*^*2*^-test). SNPs deviating from HWE (p < 0.05) were excluded from further analysis. We inferred haplotypes for combinations of SNPs using the program PHASE, version 2.1 [41,42]. In brief, the PHASE algorithm implements a Bayesian statistical method for reconstructing haplotypes from observed genotype data, dealing with missing data by imputing missing genotypes. The program constructs diplotypes for each individual with probability estimates for each diplotype. Weights, from the probability estimates derived from PHASE, were implemented in the analyses as described by French et al. [43].

SNPs were analyzed as categorical variables with two degrees of freedom, assuming a general model of inheritance. To correct for multiple comparisons, we estimated the number of independent tests within each candidate gene, taking into account the correlation between SNPs by estimating the composite linkage disequilibrium (LD) correlation matrix from the SNP data, as described by Gao et al. [44]. Next, *p*-values were adjusted according to the number of independent tests derived from the correlation matrix using Sidák corrections [45]. Haplotype analyses were performed for previously described haplotypes in a univariate model, comparing the risk haplotype against all other haplotypes by including only the risk haplotype in the Cox model. Additionally, we evaluated all common haplotypes within *FLAP* and *LTA4-H* using a multivariate model. In this model, the most common haplotype was selected as reference and all common haplotype combinations (frequencies > 1%) derived from PHASE were compared to the common haplotype by including all haplotypes as covariates in the Cox model, except for the most common haplotype. For both the uni- and multivariate models, each haplotype was evaluated, comparing non-carriers with carriers of one or two copies of the haplotype, assuming linearity for the haplotype effect [43,46].

Measures of association were assessed using Cox proportional hazards multivariate regression models with age as the time axis. In accordance with the case-cohort design, we used a weighting scheme and robust variance estimates as described by Kalbfleisch and Lawless [47]. For the haplotype analyses, these weights were multiplied with the probability weights derived from PHASE. Analyses were conducted for the whole study cohort and separately for men and women, but the pooled analysis was considered as the primary. Participants were treated as at risk from baseline until either MI, death, emigration or end of follow-up occurred.

To address potential confounding we adjusted for traditional risk factors for MI (model A2) including smoking habits (never, former or current (<15 g/day, 15–25 g/day, >25 g/day) smoker), body mass index (kg/m^2^), waist circumference (cm), physical activity (hours/week), alcohol intake (g/day), educational level (basic school, higher education: 1–3 years or >3 years) and, for women, menopausal status (pre- or post-menopausal). All continuous variables were included in the models as restricted cubic splines with five knots. Potential confounders were selected a priori based on current knowledge of risk factors for MI. In light of our primary exposure, being genetic polymorphisms, we did not expect confounding to be of major concern.

The proportional hazards assumption was checked by visual inspection of log-log plots and by evaluation of scaled Schoenfeld residuals with no violations of the assumption. P-values (two-tailed) < 0.05 were considered statistically significant. STATA, version 14.1 (StataCorp, College Station, TX, US) was used as statistical software.

## Results

### Population characteristics

**Fig 2:**
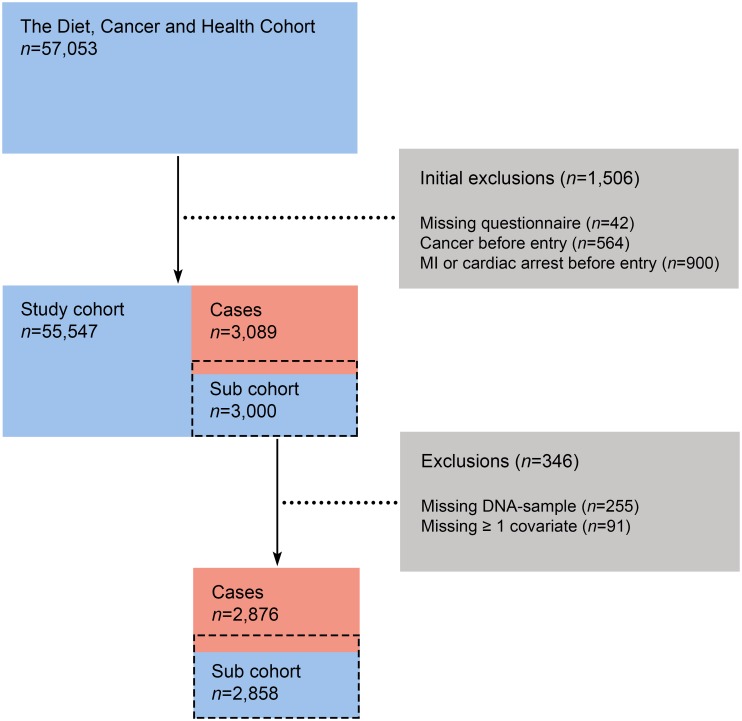
Flow chart of cohort selection process.

From an initial 160,725 invited participants, a total of 57,053 (35%) accepted the invitation, and were enrolled into the study. From these, we excluded 1,506 participants due to missing baseline questionnaires or if recorded with a cancer diagnosis or having a previous MI or cardiac arrest before baseline. In our study population we identified 3,089 cases of incident MI during a median follow-up time of 17.0 years. After case verification, we excluded subjects for whom information regarding one or more potential confounders was missing. Additionally, 255 subjects were missing DNA-samples. In total, 2,876 cases were included in the analyses (Fig 2). For individual SNPs, genotype information was missing in 57 to 148 subjects.

As expected, traditional risk factors for MI were more prevalent among cases then in the sub cohort (Table 1).

**Table 1:** Baseline characteristics of the sub cohort and cases.

	Men				Women			
Variable	Sub cohort (*n* = 1,528)		Cases (*n* = 2,048)		Sub cohort (*n* = 1,330)		Cases (*n* = 828)	
Age (years)	56.3	(51.2;63.3)	57.7	(51.7;63.9)	56.4	(51.1;63.0)	59.3	(52.4;64.2)
Physical activity (hours/week)	2.5	(0.0;8.5)	2.0	(0.0;8.0)	2.5	(0.0;8.0)	2.0	(0.0;7.0)
BMI (kg/m2)	26.4	(22.7;31.2)	26.9	(23.2;32.2)	24.6	(20.9;31.1)	25.9	(20.9;33.2)
Waist circumference (cm)	95.0	(85.0;109.0)	97.0	(86.0;112.0)	80.0	(69.0;97.0)	84.0	(70.0;102.0)
Alcohol intake (g/day)	19.4	(3.3;61.9)	18.2	(2.5;62.7)	9.3	(1.2;34.8)	6.5	(0.5;32.1)
Smoking (% (n))								
- Never smoker	25.7	(392)	18.1	(370)	42.8	(569)	27.1	(224)
- Former smoker	35.3	(540)	29.0	(594)	22.6	(301)	19.0	(157)
- <15 g/day	11.1	(169)	12.7	(259)	16.2	(215)	22.7	(188)
- 15–25 g/day	16.8	(256)	24.2	(495)	15.6	(207)	26.1	(216)
- >25 g/day	11.2	(171)	16.1	(330)	2.9	(38)	5.2	(43)
Educational level (% (n))								
- Basic school	34.0	(520)	43.3	(887)	31.7	(422)	44.6	(369)
- Higher education, 1–3 years	42.2	(645)	37.0	(758)	49.9	(664)	45.8	(379)
- Higher education, >3 years	23.8	(363)	19.7	(403)	18.4	(244)	9.7	(80)
Menopausal status (% (n))								
- Post-menopausal	N/A	N/A	N/A	N/A	59.6	(792)	69.9	(579)
- Pre-menopausal	N/A	N/A	N/A	N/A	31.1	(413)	17.0	(141)
Medical history (% (n))								
- Hypertension	14.9	(227)	22.2	(454)	17.1	(227)	31.3	(259)
- Hypercholesterolaemia	8.4	(129)	12.0	(245)	6.5	(86)	13.0	(108)
- Diabetes mellitus	3.1	(48)	5.4	(111)	1.4	(19)	4.5	(37)

Abbreviations: BMI, Body mass index.

Continuous variables are reported as medians (10th;90th percentile) and categorical variables as percent (n).

### Association between *ALOX-5* tandem-repeat polymorphism and MI

A tandem-repeat polymorphism, located close to the promoter region of *ALOX-5*, was analyzed by traditional sequencing (rs59439148). The genotype frequencies, according to the number of hexamer-repeats (‘-CCCGCC-’) for the two alleles, are presented in Table 2. The 5-repeats allele was by far, the most common allele (84.4%). Next, the 4-repeats was the most frequent variant allele observed (15.2%). Alleles with less than 4-repeats were rare (<1%). As a result of the observed allele frequencies, we analyzed the tandem repeat defining the 5-repeats allele as the wild type and alleles with less than 5-repeats as the variant alleles. Frequencies of the constructed variant and wildtype are reported in Table 3. In men, the tandem-repeat polymorphism, rs59439148, was positively associated with MI when comparing homozygous carriers of the variant with carriers of the wild type (HR = 1.63 with 95% CI: 1.06;2.52), suggesting a recessive genetic effect (S1 Table). However, no association was seen for women and the combined analyses were not statistically significant (Table 4), although the hazard ratios suggested a positive association as in men.

**Table 2:** Distribution of genotypes for the ALOX-5 promoter polymorphism, according to number of tandem repeats (5'-GGGCGG-3').

Genotype	Sub cohort		Cases	
22	-	-	-	-
23	-	-	-	-
24	2	(0.07)	2	(0.07)
25	17	(0.59)	11	(0.38)
26	-	-	-	-
33	-	-	-	-
34	3	(0.10)	1	(0.03)
35	1	(0.03)	3	(0.10)
36	-	-	-	-
44	73	(2.55)	82	(2.85)
45	717	(25.09)	679	(23.61)
46	-	-	-	-
55	2,004	(70.12)	2,039	(70.90)
56	-	-	-	-
66	-	-	1	(0.03)

Abbreviations: ALOX-5, Arachidonate 5-lipoxygenase.

Reported as number of subjects with frequencies in parentheses (%). No observations are indicated with a dash.

**Table 3:** Minor allele frequency for each SNP, selected from four candidate genes in the 5-LOX pathway.

SNP	Genomic position	Allele	Sub cohort		Cases	
***ALOX-5***						
rs12762303	10: 45373723	C/T	14.9	(833)	14.4	(809)
rs59439148	10: 4537413(2–7)	V/W	15.8	(891)	15.3	(863)
***FLAP***						
rs17222814	13: 30725416	A/G	11.0	(619)	10.6	(599)
rs4073259	13: 30732134	G/A	35.9	(2,004)	35.3	(1,983)
rs10507391	13: 30737959	A/T	32.9	(1,855)	32.4	(1,836)
rs4769874	13: 30752304	A/G	3.8	(213)	3.6	(200)
rs9551963	13: 30758410	A/C	50.3	(2,831)	48.2	(2,723)
rs9315050	13: 30761908	G/A	6.1	(342)	6.5	(366)
rs17222842	13: 30765980	A/G	10.7	(598)	9.7	(547)
***LTC4-S***						
rs730012	5: 179793637	C/A	30.4	(1,705)	31.3	(1,765)
***LTA4-H***						
rs61937881	12: 95999809	T/C	24.3	(1,349)	25.7	(1,440)
rs2660880	12: 96007474	A/G	6.8	(385)	6.8	(387)
rs6538697	12: 96009832	C/T	7.2	(408)	7.4	(419)
rs1978331	12: 96015423	C/T	38.3	(2,144)	39.9	(2,249)
rs17677715	12: 96020673	C/T	17.7	(998)	19.0	(1,076)
rs2247570	12: 96028599	G/A	29.5	(1,653)	30.8	(1,735)
rs2660898	12: 96032219	G/T	31.8	(1,783)	33.1	(1,872)
rs2540482	12: 96041102	G/A	22.5	(1,263)	22.1	(1,251)
rs2540477	12: 96043776	C/T	22.0	(1,238)	21.7	(1,228)
rs2660845	12: 96044775	G/A	26.0	(1,460)	25.6	(1,447)
rs2540475	12: 96047515	T/C	20.5	(1,159)	20.0	(1,131)

Abbreviations: SNP, Single nucleotide polymorphism; ALOX-5, Arachidonate 5-lipoxygenase; FLAP, 5-lipoxygenase activating protein; LTC4-S, Leukotriene C4 synthase; LTA4-H, Leukotriene A4 hydroxylase.

Results presented as allele frequencies (n) for the minor allele(underlined). The two SNPs (rs17216473 & rs3776944) did not display variation in our study population. Alleles correspond to the positive DNA-strand and genomic position are obtained from dbSNP, human assembly GRCh38.p2.

**Table 4:** Association of selected single nucleotide polymorphisms with incident myocardial infarction.

SNP	Genotype	Model A1*		*p*^a^	*p*^b^	Model A2**		*p*^a^	*p*^b^
***ALOX-5***									
rs12762303	T/T	1 (ref)				1 (ref)			
	C/T	0.94	(0.83;1.07)	0.34	-	0.96	(0.84;1.10)	0.55	-
	C/C	1.27	(0.87;1.86)	0.22	-	1.37	(0.91;2.05)	0.13	-
rs59439148	W/W	1 (ref)				1 (ref)			
	W/V	0.94	(0.83;1.06)	0.31	-	0.96	(0.84;1.10)	0.52	-
	V/V	1.23	(0.89;1.71)	0.21	-	1.35	(0.96;1.90)	0.09	-
***FLAP***									
rs17222814	G/G	1 (ref)				1 (ref)			
	G/A	0.91	(0.79;1.05)	0.20	0.74	0.93	(0.80;1.08)	0.34	0.92
	A/A	1.22	(0.72;2.05)	0.47	0.98	1.18	(0.66;2.10)	0.58	0.99
rs4073259	A/A	1 (ref)				1 (ref)			
	A/G	0.95	(0.84;1.07)	0.38	0.94	0.93	(0.82;1.06)	0.28	0.86
	G/G	0.96	(0.81;1.15)	0.69	>0.99	0.96	(0.80;1.17)	0.70	>0.99
rs10507391	T/T	1 (ref)				1 (ref)			
	T/A	1.00	(0.89;1.12)	0.99	>0.99	0.99	(0.87;1.12)	0.83	>0.99
	A/A	0.94	(0.78;1.14)	0.55	0.99	0.98	(0.80;1.19)	0.82	>0.99
rs4769874	G/G	1 (ref)				1 (ref)			
	G/A	0.96	(0.77;1.19)	0.72	>0.99	1.04	(0.83;1.31)	0.74	>0.99
	A/A	0.27	(0.06;1.28)	0.10	0.47	0.33	(0.07;1.61)	0.17	0.67
rs9551963	C/C	1 (ref)				1 (ref)			
	C/A	0.86	(0.75;0.98)	0.03	0.14	0.83	(0.72;0.96)	0.01	0.07
	A/A	0.85	(0.73;0.99)	0.04	0.21	0.80	(0.68;0.95)	0.01	0.05
rs9315050	A/A	1 (ref)				1 (ref)			
	A/G	1.03	(0.87;1.23)	0.71	>0.99	1.07	(0.89;1.28)	0.50	0.98
	G/G	0.66	(0.28;1.55)	0.34	0.92	0.81	(0.34;1.93)	0.63	>0.99
rs17222842	G/G	1 (ref)				1 (ref)			
	G/A	0.97	(0.85;1.12)	0.73	>0.99	0.94	(0.81;1.10)	0.45	0.97
	A/A	0.40	(0.22;0.73)	0.01	0.02	0.44	(0.24;0.82)	0.01	0.05
***LTC4-S***									
rs730012	A/A	1 (ref)				1 (ref)			
	A/C	1.11	(0.99;1.25)	0.07	-	1.08	(0.95;1.22)	0.24	-
	C/C	1.02	(0.83;1.24)	0.88	-	1.00	(0.81;1.24)	0.99	-
***LTA4-H***									
rs61937881	C/C	1 (ref)				1 (ref)			
	C/T	1.05	(0.94;1.18)	0.40	0.95	1.02	(0.90;1.16)	0.71	>0.99
	T/T	1.19	(0.94;1.50)	0.14	0.60	1.23	(0.96;1.58)	0.10	0.48
rs2660880	G/G	1 (ref)				1 (ref)			
	G/A	1.07	(0.91;1.27)	0.41	0.96	1.06	(0.89;1.28)	0.51	0.99
	A/A	0.83	(0.41;1.70)	0.61	>0.99	0.80	(0.38;1.66)	0.54	0.99
rs6538697	T/T	1 (ref)				1 (ref)			
	T/C	1.01	(0.86;1.19)	0.88	>0.99	1.04	(0.87;1.23)	0.69	>0.99
	C/C	0.99	(0.45;2.21)	0.99	>0.99	1.08	(0.48;2.43)	0.85	>0.99
rs1978331	T/T	1 (ref)				1 (ref)			
	T/C	1.06	(0.94;1.19)	0.38	0.94	1.06	(0.93;1.21)	0.38	0.94
	C/C	1.15	(0.97;1.36)	0.10	0.48	1.19	(0.99;1.43)	0.06	0.30
rs17677715	T/T	1 (ref)				1 (ref)			
	T/C	1.05	(0.93;1.18)	0.43	0.97	1.04	(0.91;1.18)	0.56	0.99
	C/C	1.25	(0.91;1.72)	0.17	0.66	1.29	(0.92;1.81)	0.14	0.59
rs2247570	A/A	1 (ref)				1 (ref)			
	A/G	1.00	(0.90;1.13)	0.94	>0.99	1.00	(0.88;1.13)	0.97	>0.99
	G/G	1.23	(1.01;1.51)	0.04	0.22	1.28	(1.03;1.59)	0.03	0.15
rs2660898	T/T	1 (ref)				1 (ref)			
	T/G	1.09	(0.97;1.22)	0.17	0.66	1.07	(0.94;1.21)	0.32	0.90
	G/G	1.09	(0.90;1.32)	0.37	0.94	1.12	(0.92;1.38)	0.26	0.84
rs2540482	A/A	1 (ref)				1 (ref)			
	A/G	0.95	(0.85;1.07)	0.38	0.94	0.95	(0.84;1.08)	0.44	0.97
	G/G	1.05	(0.81;1.36)	0.74	>0.99	0.98	(0.74;1.30)	0.89	>0.99
rs2540477	T/T	1 (ref)				1 (ref)			
	T/C	0.95	(0.85;1.07)	0.39	0.95	0.95	(0.84;1.08)	0.45	0.97
	C/C	1.08	(0.83;1.41)	0.58	0.99	1.03	(0.77;1.37)	0.85	>0.99
rs2660845	A/A	1 (ref)				1 (ref)			
	A/G	0.93	(0.83;1.05)	0.25	0.82	0.92	(0.82;1.04)	0.20	0.74
	G/G	1.04	(0.83;1.31)	0.74	>0.99	1.04	(0.81;1.33)	0.76	>0.99
rs2540475	C/C	1 (ref)				1 (ref)			
	C/T	1.00	(0.89;1.12)	0.97	>0.99	0.98	(0.86;1.11)	0.72	>0.99
	T/T	0.88	(0.67;1.17)	0.39	0.95	0.90	(0.67;1.21)	0.49	0.98

Abbreviations: SNP, Single nucleotide polymorphism; ALOX-5, Arachidonate 5-lipoxygenase; ALOX-5 AP, Arachidonate 5-lipoxygenase activating protein; LTC4-S, Leukotriene C4 synthase; LTA4-H, Leukotriene A4 hydroxylase; LD, linkage disequilibrium.

The table displays hazard ratios from a weighted cox proportional hazards model. Alleles correspond to the positive DNA-strand according to dbSNP, human assembly GRCh38.p2.

*Crude analyses. The pooled estimates are adjusted for sex.

**Adjusted analyses including sex(pooled analyses), smoking status, educational level, physical activity, BMI, waist circumference and alcohol consumption.

^a^Crude *p*-value.

^b^Adjusted *p*-value corrected for multiple testing within each candidate gene. From the composite LD correlation matrix the number of independent tests (*N*) were estimated. Using Sidák corrections, we then calculated the adjusted *p*-value as: *p*^b^ = 1-(1-*p*^a^)^*N*^.

In addition to the tandem-repeat polymorphism we also analyzed the SNP, rs12762303, that was previously shown to be in close LD with the variant and wild type allele of the tandem-repeat [29]. As anticipated, this SNP was in almost perfect LD with the tandem-repeat polymorphism (*D’* = 0.99), and the measures of association were very similar to the tandem-repeat polymorphism.

### Association of individual SNPs with MI

We genotyped 22 SNPs and examined associations with incident MI for each SNP individually. However, for two SNPs (rs17216473 and rs3776944), our assays were not able to detect the variant allele after testing two different assays on the forward strand and afterwards two assays on the reverse strand for each of the SNPs. The remaining 20 SNPs all displayed a MAF > 0.05 (Table 3) and the allele frequencies were similar to observations in other populations of European origin, according to dbSNP [39]. No SNPs deviated from the Hardy Weinberg equilibrium, when tested in the sub cohort.

In Table 4 we report hazard ratios for associations between individual SNPs and MI for the study cohort. Results are presented for both heterozygous and homozygous carriers of the variant allele compared to homozygous carriers of the wild type, assuming a general model of inheritance. Sex specific analysis are presented in the supplementary material (S1 Table).

For *FLAP*, the SNP rs9551963 was negatively associated with MI when comparing both heterozygous and homozygous carriers of the minor allele (A) with homozygous carriers of the major allele (C), suggesting a dominant genetic effect. Results were similar across sex, but only significant in men. For the combined analyses, we found a HR of 0.80 (95% CI: 0.68;0.95) for homozygous carriers of the minor allele. Rs17222842 was also negatively associated with MI, when comparing homozygous carriers of the minor allele with homozygotes of the major (HR = 0.28 (95% CI:0.12;0.63)) in men, while no association was observed in women.

Ten SNPs were successfully genotyped in *LTA4-H*. Rs2247570 was positively associated with MI in the combined analyses when comparing homozygous carriers of the minor (G) and major allele (A), HR = 1.28 (95% CI: 1.03;1.59). This relationship was consistent in both men and women, but associations were not statistically significant in the sex-stratified analyses. For rs61937881, rs1978331 and rs17677715 we found a positive association between carriers of the minor allele and MI compared with carriers of the major allele in women. However, the associations were not consistent among men, and the associations were not significant in the combined analysis for men and women.

Finally, the SNP rs730012, located in proximity to the promoter region of *LTC4-S* was not associated with MI in our data.

### Association of haplotypes with MI

Results from haplotype analyses are presented in Tables 5 and 6. First, we tested single haplotypes, previously identified in other studies, using all remaining haplotypes as reference (Table 5). In the univariate analysis we did not find any of the previously described haplotypes to be associated with MI. Next, we performed multivariate analyses including all common haplotypes (haplotype frequency > 0.01) in the Cox-model, except for the most common haplotype, that served as reference (Table 6). When comparing carriers of each variant haplotype with carriers of the most common haplotype in *FLAP*, one haplotype was negatively associated with MI (GAGAAA). However, the association was modest. For *LTA4-H* the haplotype, CGTTTATAAT, was negatively associated with incident MI.

**Table 5:** Association of selected haplotypes in FLAP and LTA4-H with incident myocardial infarction.

Haplotype	Freq. (%)	Model A1*		*p*^a^	Model A2**		*p*^a^
***FLAP***							
Hap-A (GGAT)	14.0	0.97	(0.87;1.08)	0.58	0.92	(0.82;1.04)	0.18
Hap-B (AAG)	19.8	1.02	(0.93;1.12)	0.71	1.02	(0.92;1.13)	0.71
***LTA4-H***							
Hap-K (CGTTTATGGC)	14.5	0.94	(0.85;1.05)	0.28	0.91	(0.82;1.02)	0.12
Hap-E (CCTGAA)	7.6	1.05	(0.91;1.21)	0.49	1.08	(0.93;1.26)	0.33

Abbreviations: FLAP, 5-lipoxygenase activating protein; LTA4-H, Leukotriene A4 hydroxylase.

The table displays hazard ratios from a weighted cox proportional hazards model. Haplotypes were defined as follows: Hap-A (rs17222814(G), rs4769874(G), rs9551963(A), rs10507391(T)), Hap-B (rs10507391(A), rs9315050(A), rs17222842(G)), Hap-K (rs61937881(C), rs2660880(G), rs6538697(T), rs1978331(T), rs17677715(T), rs2247570(A), rs2660898(T), rs2540482(G), rs2660845(G), rs2540475(C)), Hap-E (rs61937881(C), rs1978331(C), rs17677715(T), rs2660898(G), rs2540482(A), rs2660845(A)). Alleles correspond to the positive DNA-strand according to dbSNP, human assembly GRCh38.p2.

*Crude analyses. The pooled estimates are adjusted for sex.

**Adjusted analyses including sex(pooled analyses), smoking status, educational level, physical activity, BMI, waist circumference and alcohol consumption.

^a^Unadjusted *p*-value.

**Table 6:** Multivariate test of haplotypes in FLAP and LTA4-H and association with incident myocardial infarction.

Haplotype	Freq. (%)	Model A1*		*p*^a^	Model A2**		*p*^a^
***FLAP***							
GTGCAG	40.6	1 (ref)			1 (ref)		
GAGAAG	16.1	0.95	(0.85;1.06)	0.35	0.94	(0.83;1.06)	0.29
GAGAAA	8.4	0.89	(0.77;1.02)	0.09	0.86	(0.74;0.99)	0.04
GAGCAG	3.7	1.19	(0.99;1.44)	0.07	1.18	(0.96;1.44)	0.12
GAACGG	3.5	0.88	(0.72;1.08)	0.23	0.96	(0.77;1.19)	0.71
GTGAAG	12.4	0.96	(0.85;1.08)	0.50	0.90	(0.79;1.02)	0.11
GTGAAA	1.7	0.82	(0.62;1.08)	0.17	0.85	(0.64;1.13)	0.26
GTGCGG	1.7	0.97	(0.73;1.29)	0.83	0.92	(0.67;1.26)	0.59
ATGAAG	10.6	0.92	(0.80;1.05)	0.21	0.92	(0.80;1.06)	0.27
***LTA4-H***							
CGTTTATAAC	43.7	1 (ref)			1 (ref)		
CGTTTATGGC	14.5	0.96	(0.86;1.07)	0.46	0.93	(0.82;1.05)	0.25
CGTTTATAAT	2.0	0.74	(0.57;0.96)	0.03	0.75	(0.56;0.99)	0.04
CGTCTGTAGC	3.1	1.03	(0.82;1.29)	0.81	1.03	(0.81;1.32)	0.80
CGTCTGTAAC	1.7	0.94	(0.70;1.26)	0.68	1.03	(0.76;1.40)	0.84
CGCCTAGAAC	5.5	1.04	(0.88;1.24)	0.63	1.04	(0.87;1.25)	0.65
CGCCTAGAAT	1.4	0.96	(0.70;1.31)	0.80	1.01	(0.73;1.39)	0.97
TGTCCGGGGC	6.2	1.12	(0.95;1.32)	0.19	1.16	(0.97;1.38)	0.10
TGTCCGGAAC	1.7	1.12	(0.86;1.47)	0.40	1.10	(0.83;1.45)	0.51
TGTCCGGAAT	9.7	1.05	(0.92;1.20)	0.49	1.03	(0.89;1.19)	0.69
TATCTGGAAT	5.5	1.00	(0.84;1.18)	>0.99	1.00	(0.83;1.20)	0.99

Abbreviations: FLAP, 5-lipoxygenase activating protein; LTA4-H, Leukotriene A4 hydroxylase.

The table displays hazard ratios from a weighted cox proportional hazards model. All haplotypes with a frequency > 0.01 were included in the model except for the most common haplotype that represented the reference. Haplotypes were constructed from the following SNPs in order: rs17222814, rs10507391, rs4769874, rs9551963, rs9315050, rs17222842 (FLAP) and rs61937881, rs2660880, rs6538697, rs1978331, rs17677715, rs2247570, rs2660898, rs2540482, rs2660845, rs2540475 (LTA4-H). Alleles correspond to the positive DNA-strand according to dbSNP, human assembly GRCh38.p2.

*Crude analyses. The pooled estimates are adjusted for sex.

**Adjusted analyses including sex(pooled analyses), smoking status, educational level, physical activity, BMI, waist circumferrence and alcohol consumption.

^a^Unadjusted *p*-value.

LD maps produced from the Haploview software showed that most, but not all the selected SNPs within *FLAP* and *LTA4-H*, were in high LD with one another. This raises the possibility that some degree of recombination within the haplotype blocks had occurred in our population (S1 Fig).

## Discussion

In the large Danish Diet, Cancer and Health cohort we undertook a case-cohort study, investigating the association between 20 pre-selected SNPs and incident MI. Single SNP analyses identified two markers in *FLAP* that were negatively associated with MI (rs9551963 and rs17222842), and for *LTA4-H*, rs2247570 was positively associated with MI. However, only rs17222842 remained significantly associated with MI after adjusting for multiple testing. The tandem repeat polymorphism in *ALOX-5*, rs59439148, was positively associated with MI for men, while no association could be demonstrated in women. Furthermore, rs12762303 was in almost perfect LD with rs59439148. Finally, we performed haplotype analyses testing the association between previously reported haplotypes and MI. No significant associations were found.

In general, the associations of both single SNPs and haplotypes with MI were modest, and for some markers the results differed between men and women. A frequent concern in genetic association studies is the problem of multiple comparisons which raises the possibility of false positive discoveries (type I errors). We addressed this issue by limiting the number of SNP markers. Secondly, the number of statistical tests were minimized, e.g. for the single SNP analysis we tested the association with MI, assuming a general model of inheritance and refrained from testing several specific models (e.g. dominant and recessive models). Some genetic association studies also adjust the significance threshold for multiple comparisons. A frequently used method is the Bonferroni correction which adjusts the significance level by the number of individual tests for each hypothesis. However, some authors have criticized this method for being too conservative, and thereby introducing false negative discoveries (type II errors) [48]. This is particularly of concern when testing multiple SNP-markers that are tightly correlated, and therefore not independent [44]. Accordingly, we used the method described by Gao et al. [44] to correct for multiple comparisons, taking into account the possible correlation between SNP-markers. We chose to report both the adjusted and the unadjusted *p*-values in Table 4. While, in our opinion, the unadjusted *p*-values represents the most clear interpretation of the data, they might be over optimistic and the results should be interpreted cautiously along with the following limitations and strengths to the study design.

### Strengths and limitations

This study was based on a large prospective cohort study, and holds the advantages of the prospective design. There was a limited loss to follow-up and the assessment of outcome data was thorough and complete. The ethnicity of the study population was homogenous and all participants were of Caucasian ancestry. Furthermore, information on several dietary factors, lifestyle and other risk factors for MI was collected from the participants at baseline, allowing us to adjust for potential confounding. A priori, we did not expect confounding to be a major issue in this study, since the inherited genotype is subject to the principles of Mendelian randomization. However, some evidence suggests that epigenetics, including environmental factors, may influence on the expression of genes. We present the results for the crude model and an adjusted model (A2) including the most important risk factors for MI to address potential confounding. Furthermore, gender was not expected to biologically modify the associations between genotype and MI substantially, thus we considered the pooled analyses including both men and women to be the primary analyses. Secondary analyses, for men and women separately, were included as supplementary tables.

This study also had limitations. The selection of SNPs to cover the variation in each candidate gene was mostly based on findings by other studies and the results from genome wide scans, using a large number of genetic markers. While this method has proven to be highly effective in limiting the number of SNPs required for genotyping and the number of statistical tests to be performed, this method did not cover all common genetic variation within the candidate genes. Therefore, we cannot completely rule out that our study failed to capture all genetic variants that may be associated with our outcome. Two SNPs that were pre-selected could not be successfully genotyped, and despite our efforts, four different assays were not able to detect the minor allele for these two SNPs (rs17216473 and rs3776944).

Even though age was evenly distributed among men and women there were few female cases, which made the confidence intervals wider for measures of association when analysing data in women, and in particular for rare polymorphisms. The median follow-up was 17.0 years, and dietary measures were not assessed during the study period. A long follow-up period allowed us to accumulate more outcome events, but subjects might change their lifestyle and habits over time. Furthermore, changes in standard medical care and public awareness of disease prevention might influence the participants’ risk profile and limit our ability to address confounding. It is however unlikely that these changes affect measures of association for genotypes and confounding was a minor concern in this study.

### General discussion

Since the initial findings by Dwyer et al. [15] concerning *ALOX-5*, and later the deCODE group identifying *FLAP* [21] and *LTA4-H* [31] as important genes in the 5-LOX pathway, a number of studies have been undertaken in effort to replicate and add evidence to these studies. The *ALOX-5* tandem repeat polymorphism (rs59439148) was first found to be associated with higher intima-media thickness by Dwyer et al. but replications on cases with MI and coronary artery disease verified by angiography, more clinically relevant endpoints, have yielded conflicting results. Notably, two independent case-control studies on MI patients with Northern European origin (Caucasians) did not support an association between variant alleles and MI for this polymorphism [17,18]. Another study in a mixed population of Caucasians and African-Americans demonstrated a positive association, only in African-Americans [19]. Generally, studies were small and no studies reported data on men and women separately. In the present study, we found a positive association between carriers of two variant alleles and MI, but interestingly, the association was only present among men while there was no association among women. The pooled analysis showed the same association as for men, but the test was not statistically significant. While we have no apparent biological explanation for the differences between men and women, these inconsistencies may either be explained by random variations in the small case-group (women) or by modification of the associations by gender. To our knowledge, no previous study has presented data for men and women separately and we have no data to compare to our own findings. Other SNPs have been explored in *ALOX-5* [19,29] but only rs59439148 and SNPs linked to this polymorphism has been associated with atherosclerosis traits. We confirmed the findings by Assimes et al. [29], that rs12762303 was in near perfect LD with rs59439148 and measures of association were similar for these two polymorphisms.

Looking at *FLAP*, Helgadottir et al. [21] identified two haplotypes, Hap-A and Hap-B, that was associated with risk of MI in carriers of Hap-A compared to non-carriers in an Icelandic population, while the same was true for Hap-B in a British population. The results were later replicated in a Scottish cohort confirming a positive association for Hap-A, but not Hap-B, with ischemic stroke [22]. In the present study, we could not confirm the association with MI for Hap-A or -B. However, we found two individual SNPs within Hap-A and -B to be associated with MI when comparing homozygous carriers of the minor allele vs. the major allele (rs9551963 and rs17222842). Other studies have found an association between individual SNPs and MI in these haplotype blocks, but no consensus in favor of a certain SNP has been agreed upon.

Finally, we analyzed ten SNPs covering a haplotype block in LTA4-H. Again, as for FLAP we found some of the individual SNPs to be associated with incident MI but when analyzing the previously reported haplotype (Hap-K) we did not find any association with MI in our population.

Additionally, we performed a global haplotype analyses for *FLAP* and *LTA4-H* SNPs, in order to explore new and unique haplotype combinations in our cohort. In these analyses we included all common haplotypes inferred to our cohort in a multivariate model using the most common haplotype as the reference haplotype. By this method we found sporadic associations between haplotypes and MI but the associations were modest. This method of analyzing haplotypes in a multivariate model is well established, but never the less, it has not been explored in previous studies on 5-LOX genes.

In the following decade since the deCODE studies, a number of large genome wide association studies (GWAS) exploring the role of common SNPs in MI has been conducted [49,50]. Notably, none of these studies have highlighted SNPs in the 5-LOX genes investigated in this study as important risk variants in MI. However, while these large scale studies have the advantage of covering the whole genome, they are not ideal in more specific hypothesis testing involving specific pathway genes. In particular, the huge number of SNPs included in GWAS implies rigorous adjustments of the significance level, increasing the risk of false negative discoveries. We searched the publicly available database from three large GWAS, provided by the CARDIoGRAMplusC4D Consortium [51], to compare our SNPs to the findings from these large studies. None of the SNPs that were associated with MI in our study were analyzed in the GWAS, and no direct comparison was possible.

In this context, the 5-LOX pathway have been linked to atherosclerosis and MI in a variety of studies. The key mechanism linking the 5-LOX pathway to atherosclerosis lies in the formation of LT’s and their bioactive properties. Notably, the expression of multiple 5-LOX enzymes has been linked to human atherosclerotic plaques and plaque development [12,13]. Furthermore, LT’s are known to exert several pro-inflammatory effects including increased vascular permeability and chemo-attraction of monocytes [3,4]. Genetic association studies, mainly candidate gene studies, have also provided some evidence of a link between the 5-LOX pathway and atherosclerotic disease, which is supported by the present study. However, despite a number of studies performed it has not been possible to identify and confirm a functional polymorphism in most of the candidate genes, except for the tandem repeat polymorphism in *ALOX-5* (rs59439148), and the studies performed have been heterogeneous regarding design and results. These inconsistencies have led some authors to propose a “pathway approach”, taking into account that each step of the 5-LOX pathway may have a small influence on the outcome that can be detected when all steps are considered together. Crosslin et al. [32] examined how the expression of 5-LOX genes depended on the genotype of rs10507391, while another study found significant gene-gene interaction between a selected SNP from each of three 5-LOX genes [35]. Other studies have investigated the possibility that substrates of the 5-LOX pathway might interact with genetic variants in a complex environment where the effect of the genetic variant is dependent on substrate availability [16,52].

### Conclusion

In this study we found single SNPs in three out of four candidate genes to be associated with incident MI, collectively suggesting that the 5-LOX pathway may play a role in MI. However, the associations were modest and only one SNP (rs17222842) remained significantly associated with MI after correcting for multiple testing. Association between MI and previously reported haplotypes, Hap-A, -B, -E and -K could not be confirmed.

## Supporting Information

S1 Fig Linkage disequilibrium (LD) plots. (TIF)

S1 Table Association of selected single nucleotide polymorphisms with incident myocardial infarction (sex-stratified analyses). (PDF)
