# Supplementary figures and images for: Common Polymorphisms in the 5-Lipoxygenase Pathway and Risk of Incident Myocardial Infarction: A Danish Case-Cohort Study
Source: PLoS One. 2016 Nov 28;11(11):e0167217. doi: 10.1371/journal.pone.0167217 (PMC5125697; doi:10.1371/journal.pone.0167217)

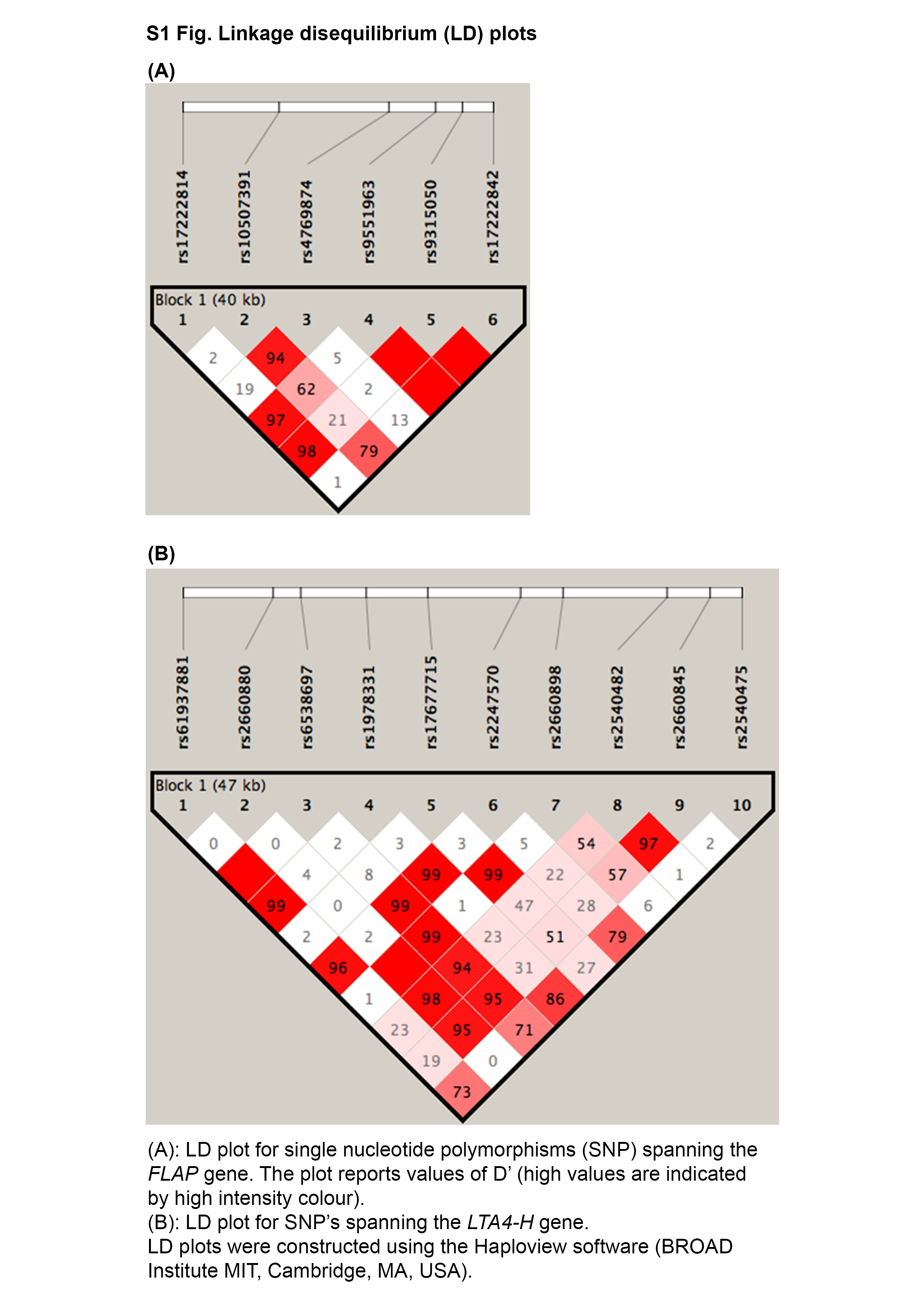

Supplement: S1 Fig — (TIF) [file pone.0167217.s001.tif]
