# Supplementary material for: Common Polymorphisms in the 5-Lipoxygenase Pathway and Risk of Incident Myocardial Infarction: A Danish Case-Cohort Study
Source: PLoS One. 2016 Nov 28;11(11):e0167217. doi: 10.1371/journal.pone.0167217 (PMC5125697; doi:10.1371/journal.pone.0167217)
Supplement: S1 Table — (PDF) [file pone.0167217.s002.pdf]

S1 Table. Association of selected single nucleotide polymorphisms with incident myocardial infarction (sex-stratified analyses).

		Men								Women							
**SNP**	**Genotype**	**Model A1***				**Model A2****				**Model A1***				**Model A2****			
ALOX-5				p^a	p^b			p^a	p^b			p^a	p^b			p^a	p^b
rs12762303	T/T	1 (ref)				1 (ref)				1 (ref)				1 (ref)			
	C/T	0.99	(0.85;1.16)	0.90	-	1.00	(0.84;1.18)	>0.99	-	0.83	(0.67;1.02)	0.08	-	0.83	(0.66;1.05)	0.12	-
	C/C	1.46	(0.89;2.39)	0.13	-	1.71	(1.03;2.84)	0.04	-	0.93	(0.51;1.71)	0.82	-	0.91	(0.45;1.85)	0.80	-
rs59439148	W/W	1 (ref)				1 (ref)				1 (ref)				1 (ref)			
	W/V	0.98	(0.84;1.15)	0.84	-	1.01	(0.85;1.19)	0.94	-	0.83	(0.67;1.03)	0.08	-	0.81	(0.65;1.03)	0.08	-
	V/V	1.45	(0.95;2.20)	0.09	-	1.63	(1.06;2.52)	0.03	-	0.86	(0.51;1.46)	0.59	-	0.91	(0.50;1.65)	0.75	-
FLAP																	
rs17222814	G/G	1 (ref)				1 (ref)				1 (ref)				1 (ref)			
	G/A	0.90	(0.75;1.06)	0.21	0.76	0.92	(0.77;1.10)	0.36	0.93	0.98	(0.78;1.23)	0.85	>0.99	0.98	(0.76;1.26)	0.85	>0.99
	A/A	1.24	(0.64;2.42)	0.52	0.99	1.14	(0.56;2.30)	0.72	>0.99	1.26	(0.57;2.80)	0.57	0.99	1.34	(0.49;3.67)	0.57	0.99
rs4073259	A/A	1 (ref)				1 (ref)				1 (ref)				1 (ref)			
	A/G	0.96	(0.83;1.12)	0.63	>0.99	0.94	(0.81;1.10)	0.48	0.98	0.90	(0.74;1.09)	0.28	0.86	0.87	(0.70;1.08)	0.21	0.76
	G/G	0.98	(0.79;1.21)	0.85	>0.99	0.97	(0.78;1.22)	0.83	>0.99	0.94	(0.70;1.26)	0.66	>0.99	0.95	(0.68;1.33)	0.77	>0.99
rs10507391	T/T	1 (ref)				1 (ref)				1 (ref)				1 (ref)			
	T/A	1.02	(0.89;1.18)	0.75	>0.99	1.01	(0.86;1.17)	0.94	>0.99	0.94	(0.78;1.14)	0.53	0.99	0.91	(0.73;1.12)	0.37	0.94
	A/A	0.97	(0.78;1.21)	0.79	>0.99	1.01	(0.79;1.28)	0.95	>0.99	0.90	(0.66;1.23)	0.52	0.99	0.93	(0.66;1.33)	0.70	>0.99
rs4769874	G/G	1 (ref)				1 (ref)				1 (ref)				1 (ref)			
	G/A	0.97	(0.75;1.25)	0.80	>0.99	1.03	(0.78;1.35)	0.86	>0.99	0.95	(0.65;1.38)	0.77	>0.99	1.02	(0.68;1.53)	0.94	>0.99
	A/A	0.69	(0.10;4.76)	0.71	>0.99	1.14	(0.16;8.06)	0.90	>0.99	-	-	-	-	-	-	-	-
rs9551963	C/C	1 (ref)				1 (ref)				1 (ref)				1 (ref)			
	C/A	0.86	(0.73;1.02)	0.08	0.39	0.83	(0.70;0.99)	0.04	0.21	0.86	(0.69;1.06)	0.16	0.65	0.84	(0.65;1.07)	0.16	0.65
	A/A	0.83	(0.69;1.00)	0.06	0.29	0.78	(0.64;0.96)	0.02	0.09	0.92	(0.71;1.18)	0.50	0.98	0.86	(0.65;1.15)	0.31	0.89
rs9315050	A/A	1 (ref)				1 (ref)				1 (ref)				1 (ref)			
	A/G	1.01	(0.82;1.24)	0.94	>0.99	1.04	(0.83;1.29)	0.74	>0.99	1.10	(0.82;1.47)	0.52	0.99	1.17	(0.85;1.61)	0.34	0.92
	G/G	1.06	(0.36;3.10)	0.92	>0.99	1.42	(0.47;4.32)	0.53	0.99	0.16	(0.02;1.28)	0.08	0.41	0.16	(0.02;1.45)	0.10	0.48
rs17222842	G/G	1 (ref)				1 (ref)				1 (ref)				1 (ref)			
	G/A	0.98	(0.82;1.17)	0.80	>0.99	0.98	(0.81;1.18)	0.83	>0.99	0.97	(0.77;1.22)	0.79	>0.99	0.93	(0.72;1.20)	0.57	0.99
	A/A	0.27	(0.12;0.59)	0.00	0.01	0.28	(0.12;0.63)	0.00	0.01	1.00	(0.41;2.43)	0.99	>0.99	1.01	(0.39;2.60)	0.99	>0.99
LTC4-S																	
rs730012	A/A	1 (ref)				1 (ref)				1 (ref)				1 (ref)			
	A/C	1.12	(0.97;1.29)	0.11	-	1.11	(0.95;1.29)	0.19	-	1.09	(0.90;1.32)	0.37	-	1.00	(0.81;1.24)	>0.99	-
	C/C	1.00	(0.78;1.27)	0.98	-	0.98	(0.76;1.27)	0.91	-	1.07	(0.78;1.48)	0.67	-	1.10	(0.76;1.59)	0.61	-
LTA4-H																	
rs61937881	C/C	1 (ref)				1 (ref)				1 (ref)				1 (ref)			
	C/T	0.99	(0.86;1.15)	0.93	>0.99	0.96	(0.82;1.12)	0.59	>0.99	1.21	(1.00;1.46)	0.05	0.26	1.25	(1.01;1.55)	0.04	0.21
	T/T	1.16	(0.88;1.55)	0.30	0.88	1.16	(0.86;1.58)	0.33	0.91	1.24	(0.85;1.81)	0.26	0.84	1.42	(0.95;2.13)	0.09	0.42

rs2660880	G/G	1 (ref)				1 (ref)				1 (ref)				1 (ref)			
	G/A	1.10	(0.89;1.35)	0.40	0.95	1.09	(0.87;1.37)	0.45	0.97	1.03	(0.79;1.34)	0.83	>0.99	0.99	(0.72;1.34)	0.93	>0.99
	A/A	0.57	(0.23;1.43)	0.23	0.80	0.53	(0.21;1.37)	0.19	0.72	1.53	(0.50;4.67)	0.45	0.97	1.56	(0.46;5.23)	0.48	0.98
rs6538697	T/T	1 (ref)				1 (ref)				1 (ref)				1 (ref)			
	T/C	0.97	(0.80;1.18)	0.76	>0.99	1.00	(0.81;1.24)	0.98	>0.99	1.12	(0.87;1.44)	0.40	0.95	1.04	(0.77;1.39)	0.82	>0.99
	C/C	0.98	(0.39;2.45)	0.97	>0.99	1.00	(0.39;2.57)	>0.99	>0.99	0.97	(0.21;4.53)	0.97	>0.99	1.15	(0.23;5.80)	0.87	>0.99
rs1978331	T/T	1 (ref)				1 (ref)				1 (ref)				1 (ref)			
	T/C	0.99	(0.86;1.15)	0.94	>0.99	0.98	(0.83;1.14)	0.76	>0.99	1.22	(1.00;1.49)	0.05	0.26	1.30	(1.04;1.62)	0.02	0.12
	C/C	1.12	(0.91;1.37)	0.30	0.88	1.14	(0.92;1.43)	0.24	0.80	1.24	(0.94;1.63)	0.13	0.56	1.27	(0.93;1.74)	0.13	0.56
rs17677715	T/T	1 (ref)				1 (ref)				1 (ref)				1 (ref)			
	T/C	1.00	(0.86;1.15)	0.95	>0.99	0.96	(0.82;1.13)	0.62	>0.99	1.19	(0.98;1.44)	0.09	0.42	1.32	(1.06;1.65)	0.01	0.08
	C/C	1.26	(0.85;1.86)	0.25	0.83	1.30	(0.86;1.98)	0.21	0.76	1.26	(0.75;2.12)	0.37	0.94	1.31	(0.76;2.24)	0.33	0.91
rs2247570	A/A	1 (ref)				1 (ref)				1 (ref)				1 (ref)			
	A/G	0.94	(0.82;1.09)	0.41	0.96	0.93	(0.80;1.08)	0.33	0.91	1.18	(0.98;1.43)	0.08	0.40	1.20	(0.97;1.48)	0.10	0.45
	G/G	1.28	(1.00;1.64)	0.05	0.28	1.28	(0.98;1.67)	0.07	0.35	1.11	(0.80;1.55)	0.54	0.99	1.27	(0.88;1.82)	0.20	0.74
rs2660898	T/T	1 (ref)				1 (ref)				1 (ref)				1 (ref)			
	T/G	1.04	(0.90;1.20)	0.57	0.99	1.02	(0.87;1.18)	0.84	>0.99	1.19	(0.99;1.44)	0.07	0.35	1.22	(0.98;1.50)	0.07	0.35
	G/G	1.03	(0.81;1.30)	0.82	>0.99	1.05	(0.82;1.34)	0.72	>0.99	1.26	(0.93;1.72)	0.14	0.58	1.27	(0.89;1.82)	0.18	0.70
rs2540482	A/A	1 (ref)				1 (ref)				1 (ref)				1 (ref)			
	A/G	0.92	(0.80;1.07)	0.28	0.87	0.94	(0.80;1.09)	0.40	0.95	1.00	(0.83;1.21)	0.96	>0.99	1.00	(0.81;1.23)	0.97	>0.99
	G/G	1.10	(0.80;1.52)	0.55	0.99	1.04	(0.74;1.46)	0.84	>0.99	0.88	(0.56;1.39)	0.59	>0.99	0.83	(0.50;1.37)	0.46	0.98
rs2540477	T/T	1 (ref)				1 (ref)				1 (ref)				1 (ref)			
	T/C	0.93	(0.81;1.07)	0.32	0.90	0.95	(0.81;1.10)	0.48	0.98	0.99	(0.82;1.20)	0.95	>0.99	0.96	(0.78;1.19)	0.73	>0.99
	C/C	1.13	(0.81;1.56)	0.48	0.98	1.06	(0.75;1.50)	0.75	>0.99	0.94	(0.60;1.48)	0.80	>0.99	0.94	(0.57;1.56)	0.82	>0.99
rs2660845	A/A	1 (ref)				1 (ref)				1 (ref)				1 (ref)			
	A/G	0.91	(0.79;1.04)	0.17	0.67	0.90	(0.78;1.05)	0.18	0.70	1.00	(0.83;1.21)	>0.99	>0.99	0.98	(0.79;1.20)	0.83	>0.99
	G/G	1.05	(0.79;1.39)	0.72	1.00	1.04	(0.77;1.41)	0.78	>0.99	0.98	(0.67;1.45)	0.94	>0.99	1.00	(0.65;1.55)	>0.99	>0.99
rs2540475	C/C	1 (ref)				1 (ref)				1 (ref)				1 (ref)			
	C/T	0.96	(0.83;1.11)	0.58	0.99	0.93	(0.80;1.09)	0.39	0.95	1.11	(0.92;1.35)	0.28	0.86	1.12	(0.91;1.40)	0.29	0.87
	T/T	0.94	(0.66;1.33)	0.74	>0.99	0.96	(0.67;1.38)	0.83	>0.99	0.75	(0.47;1.20)	0.23	0.78	0.79	(0.47;1.33)	0.38	0.94

Abbreviations: SNP, Single nucleotide polymorphism; ALOX-5, Arachidonate 5-lipoxygenase; ALOX-5 AP, Arachidonate 5-lipoxygenase activating protein; LTC4-S, Leukotriene C4 synthase; LTA4-H, Leukotriene A4 hydroxylase.

The table displayes hazard ratios from a weighted cox proportional hazards model. Results are presented for sex-stratified analyses. Alleles correspond to the positive DNA-strand according to dbSNP, human assembly GRCh38.p2.

*Crude analyses. The pooled estimates are adjusted for sex.

**Adjusted analyses including sex(pooled analyses), smoking status, educational level, physical activity, BMI, waist circumferrence and alcohol consumption.

[a]Crude *p*-value

[b]Adjusted *p*-value corrected for multiple testing within each candidate gene. From the composite LD correlation matrix the number of independent tests (*N*) were estimated. Using Sidák corrections, we then calculated the adjusted *p*-value as: $p^b = 1-(1-p^a)^N$.
